# Defect ferromagnetism in SnO_2_:Zn^2+^ hierarchical nanostructures: correlation between structural, electronic and magnetic properties

**DOI:** 10.1039/c9ra00455f

**Published:** 2019-01-30

**Authors:** S. Akbar, S. K. Hasanain, O. Ivashenko, M. V. Dutka, N. Akhtar, J. Th. M. De Hosson, N. Z. Ali, P. Rudolf

**Affiliations:** Zernike Institute for Advanced Materials, University of Groningen Nijenborgh 4 NL-9747AG Groningen The Netherlands sadafakbarmp@yahoo.com; Department of Physics, Quaid-i-Azam University Islamabad Pakistan; National Centre for Physics, Quaid-i-Azam University Campus 45320 Islamabad Pakistan; BAM Federal Institute for Materials Research and Testing Richard-Willstaetter-Strasse 11 Berlin Germany

## Abstract

We report on the ferromagnetism of Sn_1−*x*_Zn_*x*_O_2_ (*x* ≤ 0.1) hierarchical nanostructures with various morphologies synthesized by a solvothermal route. A room temperature ferromagnetic and paramagnetic response was observed for all compositions, with a maximum in ferromagnetism for *x* = 0.04. The ferromagnetic behaviour was found to correlate with the presence of zinc on substitutional Sn sites and with a low oxygen vacancy concentration in the samples. The morphology of the nanostructures varied with zinc concentration. The strongest ferromagnetic response was observed in nanostructures with well-formed shapes, having nanoneedles on their surfaces. These nanoneedles consist of (110) and (101) planes, which are understood to be important in stabilizing the ferromagnetic defects. At higher zinc concentration the nanostructures become eroded and agglomerated, a phenomenon accompanied with a strong decrease in their ferromagnetic response. The observed trends are explained in the light of recent computational studies that discuss the relative stability of ferromagnetic defects on various surfaces and the role of oxygen vacancies in degrading ferromagnetism *via* an increase in free electron concentration.

## Abstract 2

We report on the ferromagnetism of Sn_1−*x*_Zn_*x*_O_2_ (*x* ≤ 0.1) hierarchical nanostructures with various morphologies synthesized by a solvothermal route.

## Graphical abstract

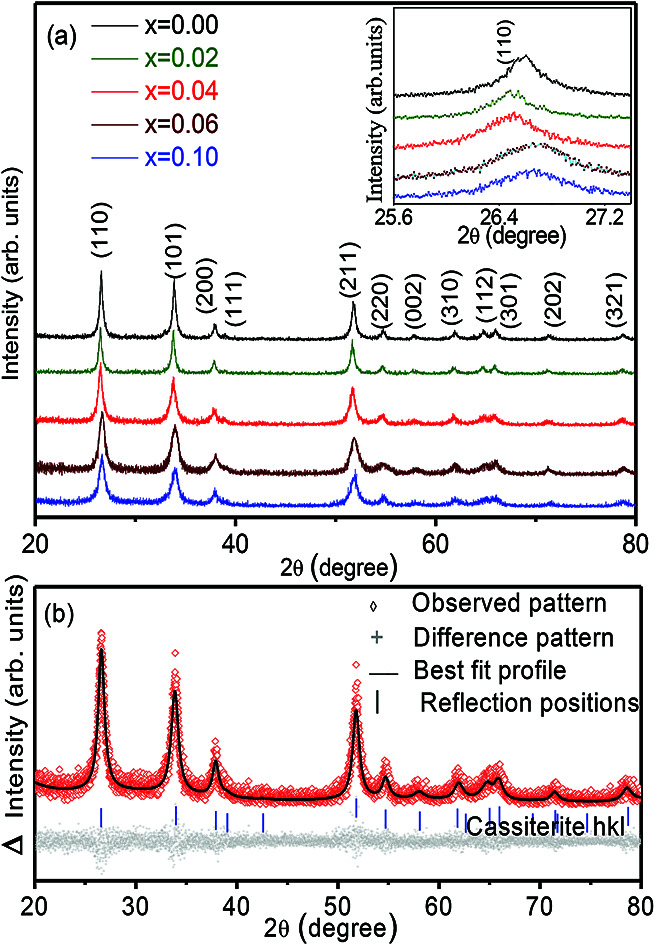

## Introduction

Stannic oxide (SnO_2_) is a wide band gap semiconductor that exhibits both relatively high electrical conductivity and insulator-like transparency in the visible range. Such properties of SnO_2_ in combination with other materials enable wide usage in optical and solar cell applications.^1,2^ The reports of room temperature ferromagnetism (FM) in both pure^3^ and non-magnetically doped SnO_2_ hold promise for increased functionalities of this system.^4–13^ FM in such systems is explained in general as a consequence of various defect-induced local structures that lead to modifications of the local charge density and the consequent polarization of the spin bands. Despite the general consensus on the role of defects, there is no unanimity on the specific defects that are important and on the mechanism whereby they are stabilized. These defects include O vacancies, Sn vacancies and cation (dopant) substitution on Sn sites. However it is also known that such defects have different formation energies that are in general very sensitive to the local atomic environment and to the ambient conditions during synthesis. Consequently the stability of these moment-supporting defects varies with dopant concentrations and their specific location in the lattice structure. In this work we studied the magnetic and morphological aspects of Zn-doped SnO_2_ and discuss the observed room temperature ferromagnetism in terms of stabilization of relevant defects in the different observed nanoscale morphologies.

Initial research efforts in this field were focused on magnetic transition metal (TM) doped SnO_2_ nanoparticles and thin films (Co, Cr, Mn, Fe, Ni and V)^14–16^ that display ferromagnetism. To avoid magnetic metal clusters or secondary phases of SnO_2_ doped with nonmagnetic (NM) elements (*e.g*. Cu and Zn),^4–6^ alkali metals (Li and K),^7–9^ alkali earth metals (Mg),^5^ non-metals (C and N),^10,11^ and poor metals (In and Ga)^12,13^ have also been studied and the FM has been reported. Density functional studies^17^ have shown that Sn vacancies (V_Sn_) are responsible for the observed giant magnetic moment (GMM) of TM-doped SnO_2_. Other computational studies^18^ describe surface magnetism induced in a C-doped (001) surface and the incorporation of Li^1+^ at (001) surface sites^19^ of SnO_2_. Surface magnetism in Cu-doped (110) surfaces in SnO_2_ thin films has also been predicted.^20^ The role of divalent zinc as a substituent for Sn^4+^ is particularly interesting due to the closeness in their respective ionic sizes on the one hand and the difference between their respective valences, on the other. Doping of SnO_2_ with Zn has been shown to induce magnetism in nanoscale systems,^6,21^ while computational studies performed on the bulk SnO_2_ system with Zn doping relate this magnetism to the native defect of tin vacancies.^22,23^ The other prevalent defects in this system, namely oxygen vacancies (V_O_) are known to weaken FM. It has been reported^24^ that divalent Zn^2+^ and Cd^2+^ ions substituting for Sn^4+^ introduce holes in the 2p orbitals of the O atoms while the induced magnetic moment arises mainly from the O 2p orbitals and is largest at the first O atom neighbouring the dopant. Hence electron deficiency at the oxygen site, whether originating from the V_Sn_ or from replacement of Sn^4+^ by Zn^2+^, leads to holes in the O 2p band and to the possible polarization of this band. According to these studies^24^ the contribution of the V_Sn_ defect to the moment itself is usually very small.

The incorporation of zinc in SnO_2_ and the stability of other common defects is however sensitive to the specific surface where these defects formed. Pushpa *et al.*^25^ have studied the formation energies and magnetic moments for various defects in the bulk, at the surface and in sub-surface layers, in both Sn-rich and O-rich conditions. In general it is easier to form both Sn and O vacancies at surfaces than in the bulk, and the (001) surface is preferred to the (110) surface. These authors have shown that although the V_Sn_ defect is magnetic for both bulk and (110) surfaces, its formation energy remains very high even at the surface. The oxygen vacancy on the other hand has no magnetic moment in the bulk nor on either of the two surfaces studied. The Zn substitutional defect (Zn_Sn_) possesses a small moment of ∼0–0.11 *μ*_B_/Zn, but generates a significant moment per unit cell (2 *μ*_B_ per cell). The formation energy for Zn_Sn_ on the (001) surfaces is about half that of the bulk, while on the (110) surfaces it is close to that of the bulk. Interestingly, the Zn atom on the (110) surface does not contribute to the induced moment directly.^25^ The moment arises from the nearest neighbour bridging oxygen and minimally from the in-plane oxygen or the Sn atom. It is further understood that while the moment arises from the polarization of the partially filled oxygen bands, the occurrence of ferromagnetism or antiferromagnetism (AFM) in these bands depends on the separation between the holes on oxygen atoms surrounding the V_Sn_ defect.

Alongside these studies of the electronic and FM/AFM properties, there are various reports of unique morphologies in SnO_2_-based systems that display hierarchical nanostructures with nanorods, nanosheets and nanoflowers at different scales, often as a function of stoichiometry.^26–28^ The formation of these nanostructures is related to differences in the growth rates of various crystallographic planes in the presence of these defects, *e.g.* oxygen vacancies, substitutional atoms, *etc.* Several previous reports^26,27,29–34^ have demonstrated that the morphologies and properties of SnO_2_ can be modified by Zn doping since incorporation of zinc into the SnO_2_ lattice modifies the local structure and the growth rate of different crystallographic planes. In particular Zn^2+^ ions in the SnO_2_ lattice inhibit the growth along the [110] direction, promoting the anisotropic growth of nanorods. Because the formation energies of the moment supporting defects (*e.g.* Sn vacancies) are different for different such surfaces or planes, the morphological and electronic properties become interrelated in this system. While this aspect may not be particularly important for bulk systems, it assumes a different significance for nanostructured materials, where preferential growth of a certain surface affects the magnitude of the magnetic moment.

In the light of the preceding discussion on the role of specific planes in lowering the formation energy of defects and stabilizing ferromagnetism, we prepared nanoparticles of Zn-doped SnO_2_. A solvothermal synthesis route was adopted that resulted in hierarchal nanoarchitectures with well-defined planar surface structures that change with Zn concentration. Alongside the structural changes the magnetic behaviour also changes and this study contributes to the deeper understanding of the observed variations in structure, morphology and magnetic property relationship.

## Experimental

Sn_1−*x*_Zn_*x*_O_2_ nanoparticles with varying Zn concentration (*x* = 0, 0.02, 0.04, 0.06, 0.10) were synthesized by a simple solvothermal method at room temperature. All chemicals were of analytical grade and used without further purification. 0.1 M of SnCl_4_·5H_2_O and Zn(NO_3_)_2_·6H_2_O were separately dissolved in 30 mL of a mixture of ethanol and deionized (DI) water (1 : 1 volume ratio). Then a 30 mL ethanol–DI water (1 : 1 volume ratio) solution containing 2.4 g NaOH was slowly added dropwise under rigorous stirring. After 30 min the ensuing mixture (pH value ∼11) was transferred into a 100 mL Teflon-lined stainless steel autoclave and kept at 160 °C for 22 h. The resulting precipitates were separated by centrifugation and washed several times with ethanol and DI water. Finally, the products were dried in air at 80 °C for several hours. NaOH is a very favourable additive for the growth of 1D SnO_2_ nanostructures, with [001] direction as the growth axis and (110) as the family of enclosing facets.^35^ Higher pH values used in the synthesis accelerate the nucleation rate, which results both in a higher nuclei concentration and in higher growth rates of nanoparticles. As discussed in detail in ref. 26, when Zn^2+^ ions are introduced into the solution, some Sn^2+^ ions are substituted, forming compound nuclei under the solvothermal conditions. Later some of these nuclei grow into nanosheet structures through aggregation^36^ and Ostwald ripening^37^ under the influence of Zn^2+^. Finally, the nanosheets aggregate to reduce the surface area and the associated surface energy.

A PANalytical X'Pert PRO X-ray diffractometer (XRD) equipped with Cu K_α_ radiation (*λ* = 1.5405 Å) was used for the structural analysis of the samples. The morphology and microstructure of the samples were investigated by Field Emission Scanning Electron Microscopy (XL30-FEI ESEM-FEG, 5k–30 kV), equipped with energy-dispersive X-ray spectroscopy (EDS) and High Resolution Transmission Electron Microscopy (HRTEM) (JEOL2010FEG operating at 200 kV). X-ray photoelectron spectroscopy (XPS) was employed to analyse the chemical composition of the prepared Zn-doped SnO_2_ nanoparticles. XPS data were collected using a Surface Science SSX-100 ESCA spectrometer equipped with a monochromatic Al K_α_ X-ray source (*hν* = 1486.6 eV) under UHV conditions. All binding energies were referred to the C 1s line at 284.6 ± 0.1 eV (stemming from adventitious carbon). Spectral analysis comprised a Shirley background subtraction and peak deconvolution employing a convolution of Gaussian and Lorentzian functions with a 90–10% ratio by a least-square fitting program (Winspec), developed in the LISE laboratory of the Facultés Universitaires Notre-Dame de la Paix, Namur, Belgium. Magnetic characterization of the samples was carried out using a Quantum Design MPMS-XL7 SQUID magnetometer.

## Result and discussion

### Structural analysis

**Fig. 1:**
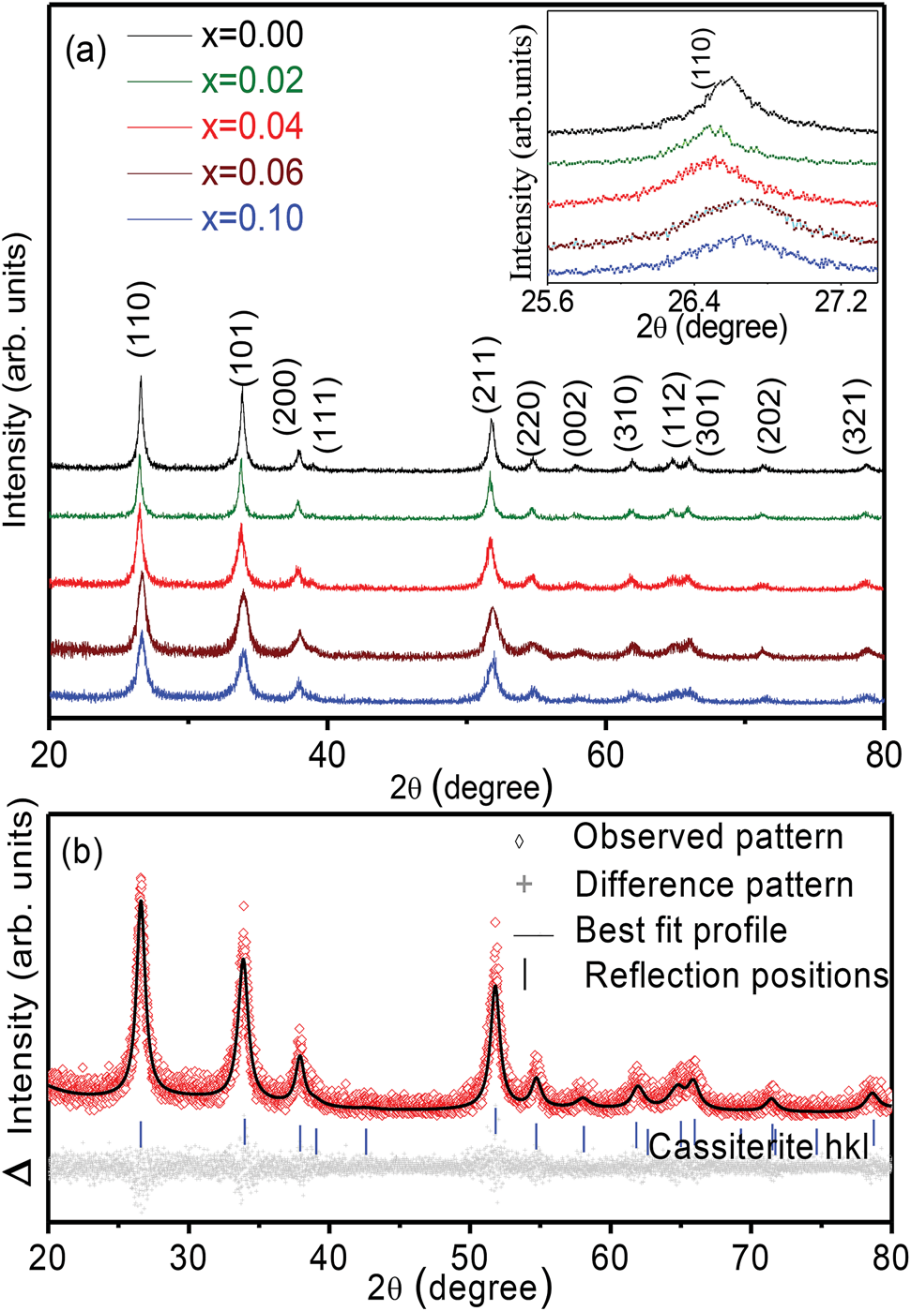
(a) XRD patterns of Sn_1−*x*_Zn_*x*_O_2_ with *x* varying between 0 and 0.10. Inset: detail of the (110) peak, which shifts with Zn addition. (b) Rietveld refinement of XRD pattern for the *x* = 0.04 composition.

Fig. 1(a) presents the X-ray diffraction (XRD) data of the undoped and the Zn-doped SnO_2_ nanoparticles. All the diffraction peaks in the pattern correspond to the tetragonal rutile structure of polycrystalline SnO_2_ (JCPDS File no. 41-1445). No phase corresponding to zinc or other zinc compounds was observed, indicating that zinc gets incorporated into the tin oxide lattice. These XRD patterns were refined with the help of the X'Pert High Score Plus software and by the Rietveld refinement technique using TOPAS program (version 4.1-Bruker AXS).^38^

**Fig. 2:**
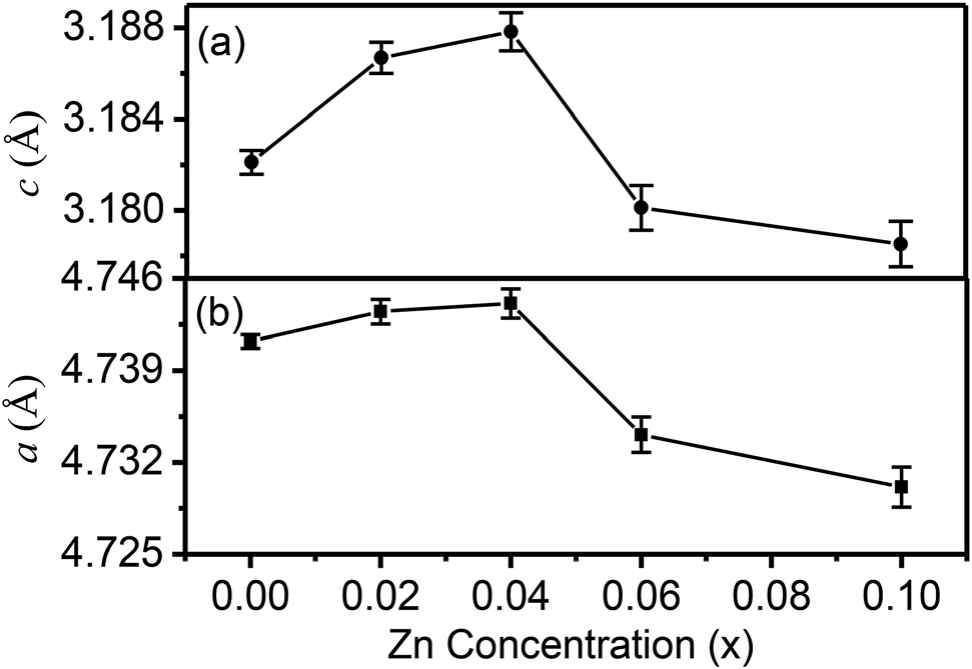
Variation of (a) lattice parameter *c* and (b) lattice parameter *a* determined from XRD as function of Zn concentration (*x*) in Sn_1−*x*_Zn_*x*_O_2_.

A typical XRD pattern along with the refinement is shown in Fig. 1(b) for the Sn_0.96_Zn_0.04_O_2_ sample. It can be clearly seen that the experimentally observed X-ray peaks accurately match with simulated pattern refined on mineral Cassiterite model in tetragonal space group *P*4_2_/*mnm*. Using the XRD data, the cell parameters *a* and *c* were calculated for different doping concentrations (*x*), and their average values are plotted in Fig. 2(a) and (b) respectively. The trend of the calculated values clearly indicates an increase in the values of lattice parameters *a* and *c* with increasing Zn concentration up to *x* = 0.04. The observed expansion could be due to interstitial occupation however we understood this expansion as substitution of Zn^2+^ with larger ionic radii of 0.74 Å at Sn^4+^ (0.69 Å) ions. This indicates that the Zn dopant atoms are accommodated substitutionally, filling tin vacancies. In addition, the substitution of Sn-ions by Zn can generate oxygen vacancies for charge compensation.^6^ Zn ions in solid solution can be excluded because we do not observe maximum full width at half maximum (FWHM) at *x* = 0.04.

For *x* > 0.04 both *a* and *c* decrease up to the highest concentration studied, namely *x* = 0.10. This decrease on substitution would also lead to a cell volume reduction since the size of Zn^2+^ is much smaller than that of O^2−^ (1.4 Å). We also note that with increasing Zn concentration, the diffraction peaks decrease in intensity and tend to become broader as shown in the inset of Fig. 1(a). The changes in intensity and full width at half maximum (FWHM) indicate that the incorporation of Zn dopants results in the deterioration of crystallinity and the decrease of grain size in Sn_1−*x*_Zn_*x*_O_2_ samples. The average grain size was estimated using the FWHM of (110) and (101) peaks based on Scherrer's equation. As the Zn concentration in Sn_1−*x*_Zn_*x*_O_2_ increases from *x* = 0.02 to *x* = 0.10, the average grain size decreases from 80.0 ± 2.1 nm to 15.0 ± 2.1 nm. This aspect will be discussed further in the context of the morphological studies on these particles.

Energy dispersive X-ray spectroscopy (EDS) allows to check the presence of any unwanted magnetic impurity within the instrumental detection limit of 1%. The analysis confirms that there are no detectable traces of magnetic impurities in the compounds.

**Fig. 3:**
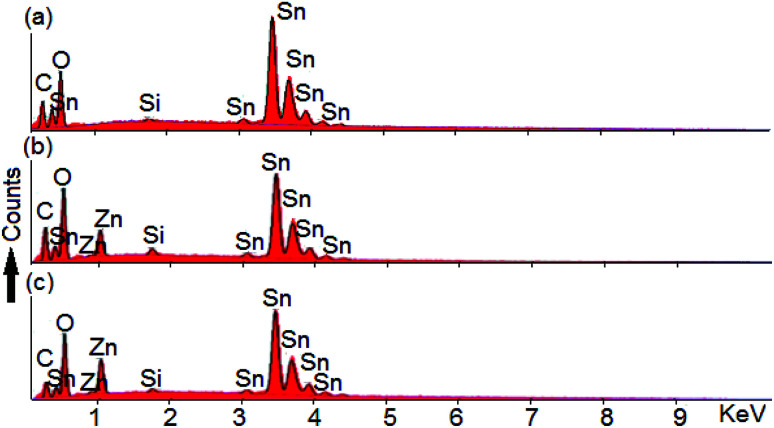
Energy-dispersive X-ray spectra of (a) undoped, (b) Sn_0.96_Zn_0.04_O_2_ and (c) Sn_0.90_Zn_0.10_O_2_ nanoparticles.

The results are shown in Fig. 3. The elemental analysis corroborates the presence of Zn, Sn and O as well as giving evidence for Si and C signals coming from the sample holder with conductive tape.

### Morphology and structure of Zn-doped SnO_2_ hierarchical architectures

**Fig. 4:**
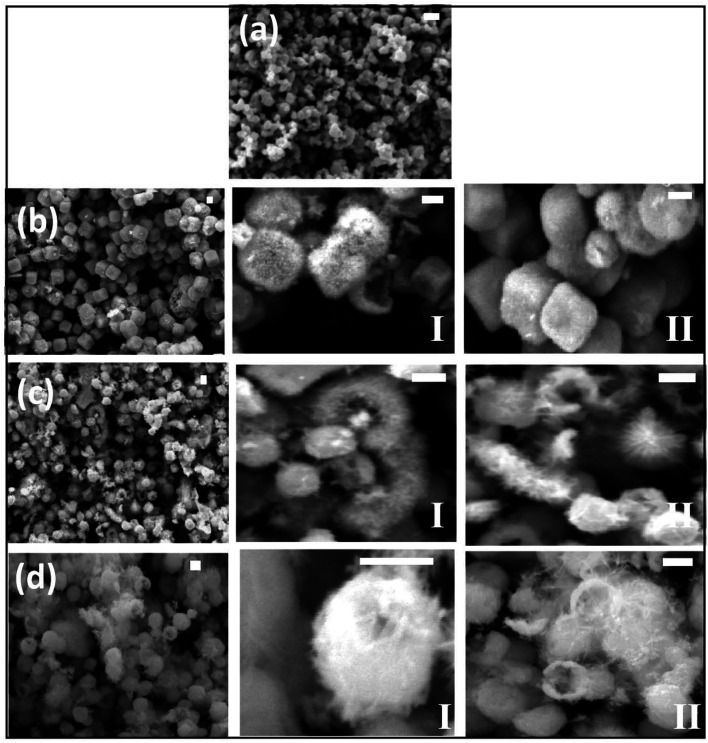
Field emission SEM micrographs of Zn_*x*_Sn_1−*x*_O_2_ nanoparticles with (a) *x* = 0.00, (b) *x* = 0.04, (c) *x* = 0.06 and (d) *x* = 0.10, where I and II represent the corresponding higher magnification micrographs. The scale bar corresponds to 500 nm in each case.

The morphology of the Zn-doped SnO_2_ with different Zn content was studied by FESEM, EDS and TEM. Fig. 4(a) shows aggregated spherical nanoparticles of undoped SnO_2_ (*x* = 0.00) with sizes in the range 50–200 nm. Fig. 4(b) reveals that with the addition of Zn (Sn_0.96_Zn_0.04_O_2_) the aggregated undoped nanospheres grow into μm-size mainly cubes, spheres and some into eroded cubes and spheres. Some of the surfaces of the Zn doped particles are covered with fine needle-like growth, while a few of the nanospheres acquired a flower like morphology.

Fig. 4(c) and (d) present images for the Sn_1−*x*_Zn_*x*_O_2_ with *x* = 0.06 and *x* = 0.10 respectively. With increasing Zn concentration the spherical and cubic particles become eroded and acquire different shapes of hierarchical structures. These include a mixture of hemi- and hollow spheres, and elongated chains of flower-type structures. We also observed that for higher zinc concentration these μm-size structures become interconnected by nanoneedles on their surfaces. For *x* = 0.10 one sees a clear erosion of the individual cubical and/or spherical structures, which now agglomerate to form a bundle of nanoflowers or different shapes, with nanoneedles on their surfaces. For further analysis of the nanoneedles, TEM and HRTEM micrographs were collected from a Sn_1−*x*_Zn_*x*_O_2_ sample with *x* = 0.04.

**Fig. 5:**
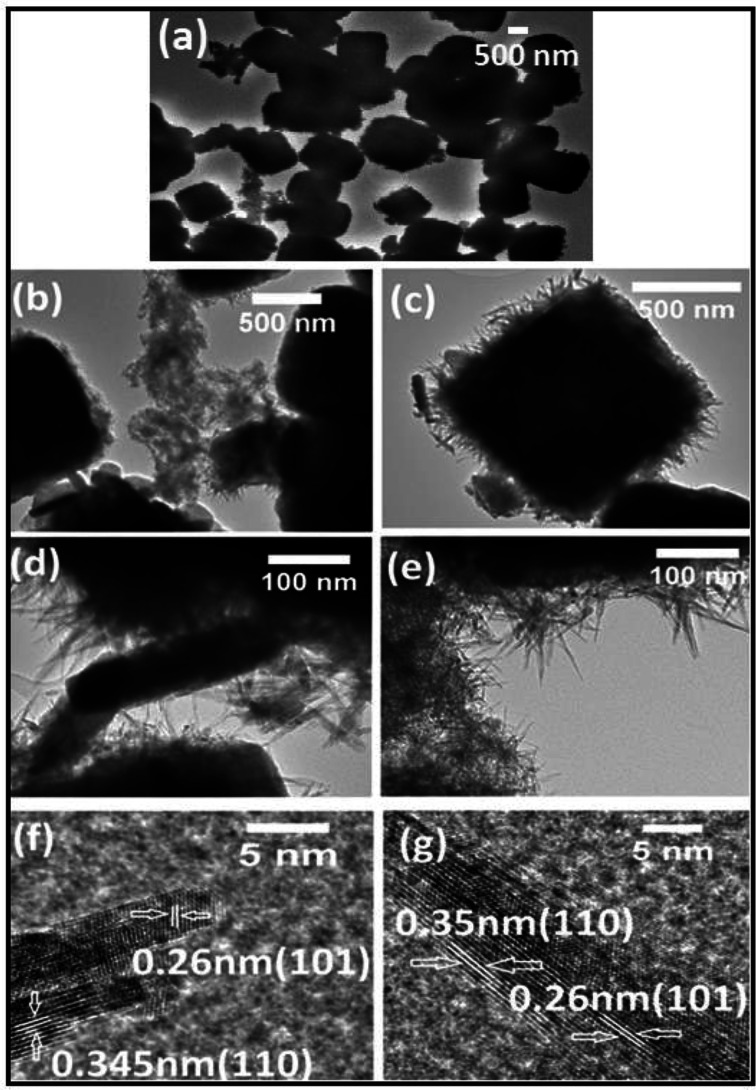
TEM (a–e) and HRTEM (f and g) images of Zn_*x*_Sn_1−*x*_O_2_ with *x* = 0.04. HRTEM focuses on the nanoneedles extending from the nanoparticle surfaces in (a–e). The (110) and (101) planes are visible.

Fig. 5(a) presents an image of this sample where μm-sized particles with mainly cubical shapes can be seen. High resolution TEM images in Fig. 5(b–e) clearly show that these μm-size particles are covered with outward growing nanoneedles, nanorods and nanostructures extending from the surface. The length of these nanoneedles is in the range 10–100 nm (Fig. 5(d) and (e)), and connecting nanorods measure about 85 nm × 280 nm (Fig. 5(d)). Fig. 5(f) and (g) show HRTEM micrographs of long and small nanoneedles. The two groups of crystallographic planes marked in the images have interplanar distances of 0.35 nm and 0.26 nm respectively. These separations match well with the (110) and (101) planes of rutile SnO_2_.

In SnO_2_ the (110) surface has the lowest surface energy, followed by the (100), (101) and (001) surfaces.^40^ Nanocrystals have a high surface-to-volume ratio and tend to aggregate to decrease the surface energy. During the initial stages of the solvothermal process, SnO_2_ spherical nanoparticles were produced with diameters in the range 50–200 nm,^41^ at higher Zn concentrations these nanoparticles aggregate for energy minimization into solid cubes with needles on the surface. It should be noted that these solid cubes and spheres are all composed of nanocrystalline particles, as shown in Fig. 5(b). A similar behaviour has been demonstrated in the preparation of other hollow structures, such as hollow Cu_2_O cubes and hollow TiO_2_ spheres.^42,43^ Furthermore, in the absence of Zn^2+^ ions, a similar morphology was not obtained and pure SnO_2_ (Fig. 4(a)) shows no evidence for nanoneedle-like growth or interconnecting nanorods. While similar nanostructures have been reported in pure SnO_2_ nanoparticles, their development in un-doped SnO_2_ appears connected to the presence of Sn^2+^ ions. In the case of Zn-doped SnO_2_, the role of Zn^2+^ as a structure directing agent has been reported^26^ and is confirmed by our results.

The substitution of Zn^2+^ for Sn^4+^ leads to doubly charged oxygen vacancies, 
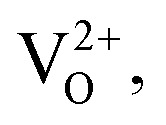
 as a charge compensation mechanism.^6^ Consequently the charge density and surface energy of various crystal faces is changed, leading to a large polarity in the growth of Zn-doped SnO_2_, which in turn yields different growth rates for different surfaces. As already mentioned the average crystallite size estimated by Scherrer's formula decreases with the addition of Zn, suggesting that the zinc dopant plays an active role in reducing the average crystallite size.

After nucleation most of the Zn^2+^ ions will segregate to surface/interface sites because of the abundant surface area available.^34^ When these ions occupy the surface sites of SnO_2_ nanocrystals, they most likely inhibit the formation of necks between particles and the coalescence of tiny SnO_2_ crystals into larger particles (microcubes and microspheres). Thus our study helps establish some important differences between the morphologies of low and high zinc concentration nanoparticle systems. Whereas Zn_*x*_Sn_1−*x*_O_2_ particles with *x* = 0.04 show well-developed isolated structures with needle-like growth on the surfaces, particles with higher Zn concentration (*x* = 0.06, 0.10) exhibit small grain sizes, erosion of shapes and agglomeration of the particles.

### X-ray photoelectron spectroscopy (XPS) analysis

**Fig. 6:**
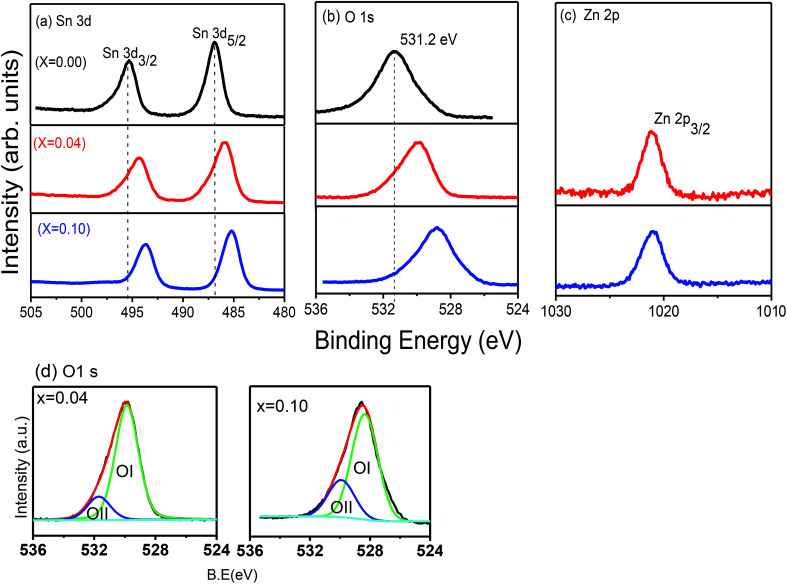
X-ray photoelectron spectra of the (a) Sn 3d, (b) O 1s and (c) Zn 2p core level regions for Zn_*x*_Sn_1−*x*_O_2_ with *x* = 0.00, 0.04 and 0.1. (d) Deconvolution of *x* = 0.04 and 0.10 of O 1s spectra. OI and OII explained in text.

The chemical composition of the hierarchical nanostructures was studied by XPS. Fig. 6(a–c) display the spectra of the Sn 3d, Zn 2p and O 1s core level regions for undoped SnO_2_, and for Zn_*x*_Sn_1−*x*_O_2_ with *x* = 0.04 and *x* = 0.10.

The undoped SnO_2_ spectrum shows Sn 3d_5/2_ and 3d_3/2_ core levels of the Sn^4+^ ions at binding energies of 486.9 and 495.3 eV respectively. For *x* = 0.04 the binding energy of Sn 3d doublet (485.9 eV and 494.3 eV) decreases by 1 eV and for *x* = 0.10 by 1.7 eV (485.2 and 493.7 eV) as compare to undoped SnO_2_. This decreases in binding energy of the Sn 3d doublet can be attributed to the presence of oxygen vacancies with addition of Zn dopant.^31,44,45^ It can be noted that at *x* = 0.10 the chemical shift in Sn 3d is not following the same trend as it is showing from *x* = 0.00 to *x* = 0.04 which could be due to the reason that at higher concentration Zn going more to bulk sites as compare to surface sites.

Fig. 6(b) displays the O 1s spectra for undoped, *x* = 0.04 and *x* = 0.10 Zn doped SnO_2_ samples. The main peak (centred at 531.2 eV for *x* = 0.00, at 529.9 eV for *x* = 0.04 and at 528.6 eV for *x* = 0.10) was assigned to the coordination of oxygen in Sn–O–Sn, while the shoulder at higher binding energy side could be ascribed to Sn–O–Zn bonds.^31,39^ The chemical shift towards lower binding energy as a function of Zn-doping can be attributed to the increasing number of V_O_. To analyse it further, Fig. 6(d) presents the fit of the O 1s spectra of *x* = 0.04 and 0.10. The deconvolution requires two components namely OI and OII. OI centred at 529.9 eV and 528.4 eV, and OII, centred at 531.7 eV and 529.9 eV correspond to lower and higher binding energy components for *x* = 0.04 and 0.10 respectively. The lower binding energy component OI is attributed to the coordination of oxygen bound to Sn atoms, whereas the higher binding energy component OII is assigned to the oxygen vacancies. The OII component is larger for *x* = 0.10 (26%) than for *x* = 0.04 (16%), indicating that the number of oxygen vacancies increases with the zinc concentration.

Fig. 6(c) displays the Zn 2p core level region where the Zn 2p_3/2_ peak appears at a binding energy of 1021.1 eV,^21,46^ confirming that Zn atoms were incorporated into the Sn lattice and form Zn–O bonds. We analysed at least three different spots on every sample and found that the Zn concentration did not vary between different spots, which indicates that the Zn concentration in these samples is homogeneously distributed. We find out Zn/Sn ratio by XPS data. For *x* = 0.04 the ratio came out as expected, however a lower number was obtained for *x* = 0.10 *i.e.* 0.05. This could be understood that Zn goes to surface first for lower concentration, however with the increasing concentration of dopants, Zn also starts going into the bulk and XPS shows lower number as being more surface sensitive technique.

It can be concluded that at low concentrations Zn substitutes more to surface sites than at bulk. It is well known in nanoparticles, that surface sites are more activated.^34,49^

### Magnetic analysis

**Fig. 7:**
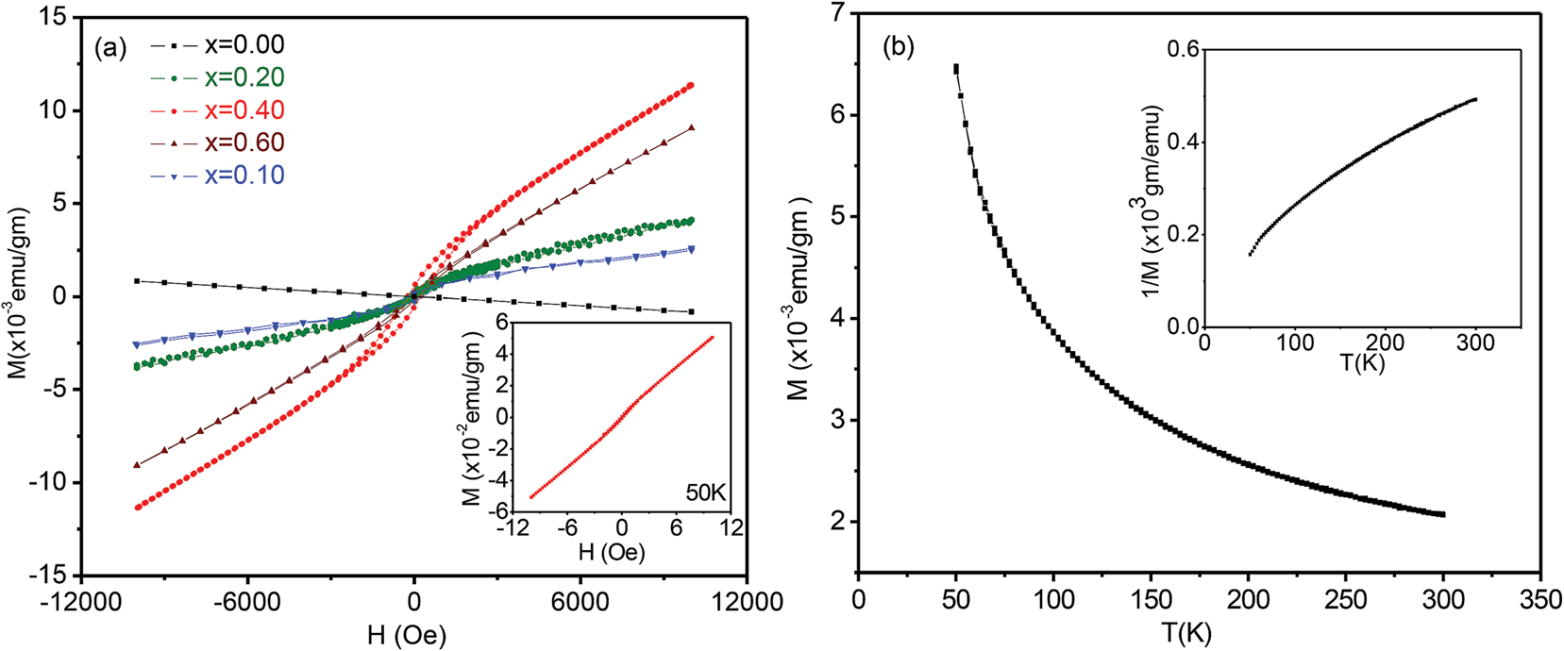
(a) Room temperature magnetization (*M*) *versus* magnetic field (*H*) of Sn_1−*x*_Zn_*x*_O_2_ with *x* = 0.00, 0.02, 0.04, 0.06 and 0.10. Inset *M vs. H* at 50 K of Sn_1−*x*_Zn_*x*_O_2_ with *x* = 0.04. (b) Magnetization *versus* temperature for Sn_1−*x*_Zn_*x*_O_2_ with *x* = 0.04 with an applied field of 1000 Oe. The inset shows 1/*M vs.* temperature, which deviates from a straight line.

Magnetization measurements *M*(*H*) were carried out at room temperature for all samples and for selected compositions at 5 or 50 K. Magnetization *versus* temperature data were also recorded for Zn_*x*_Sn_1−*x*_O_2_ with *x* = 0.04. Fig. 7(a) shows the field dependence of the magnetization *M*(*H*) for all the samples studied. Pure SnO_2_ was diamagnetic at room temperature, while at 5 K it showed paramagnetic behaviour, *i.e.* a linear dependence of the magnetization on the applied field. The magnetic moment at high fields was maximum for *x* = 0.04 and declined strongly for lower and higher Zn content. All Zn-doped SnO_2_ samples showed the presence of two contributions. Firstly, there is a non-linear or ferromagnetic component with a non-zero remanence and hysteresis at room temperature.

The *x* = 0.04 (inset Fig. 7(a)) and 0.06 compositions were also measured at low temperature and showed a strong increase in both the remanence and hysteresis. The second component of the magnetization, as is evident from Fig. 7(a), is a linearly increasing or paramagnetic component.

The magnetization data for *x* = 0.04 as a function of temperature for an applied field of 1000 Oe is shown in Fig. 7(b), while the inset shows the inverse of magnetization *versus* temperature. It is apparent from the curvature of the 1/*M versus T* that the data does not show a good Curie–Weiss behaviour. This is of course not surprising, since the full magnetization includes a ferromagnetic component in addition to the paramagnetic part.

**Fig. 8:**
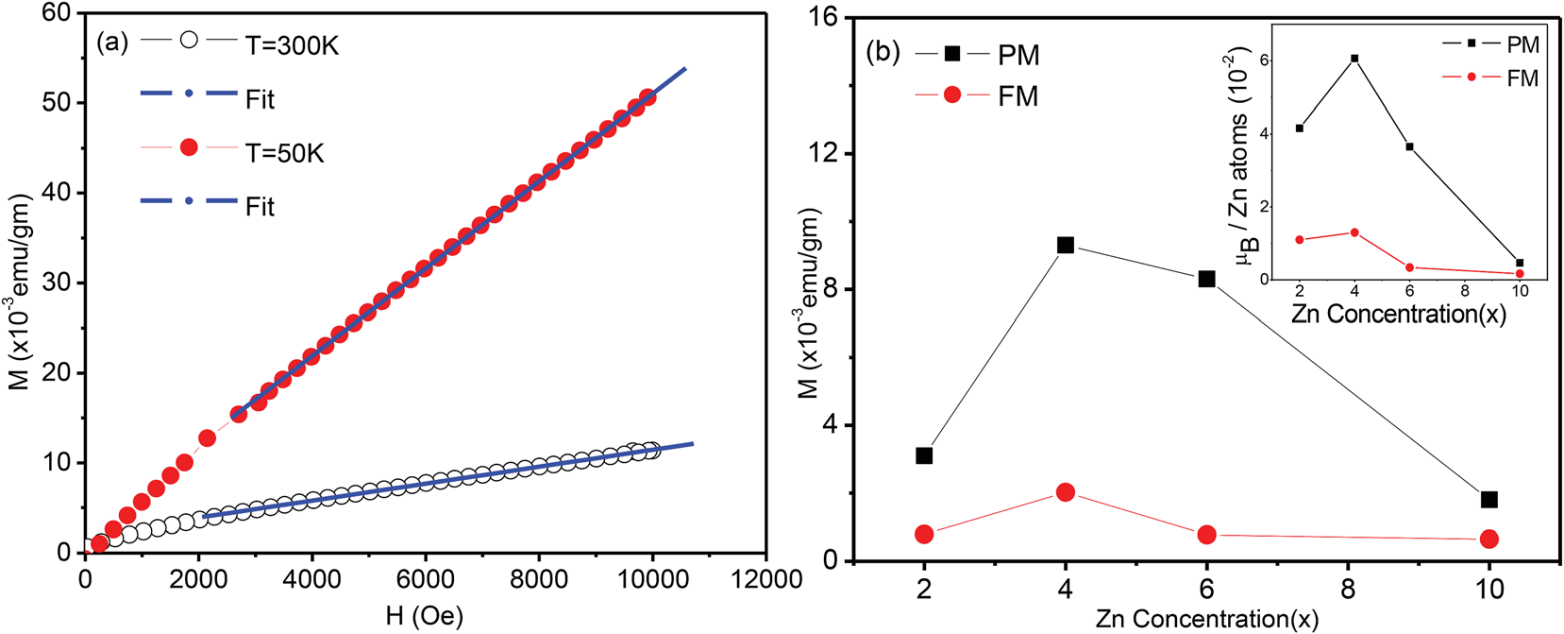
(a) Magnetization (*M*) *versus* magnetic field (*H*) of Sn_1−*x*_Zn_*x*_O_2_ with *x* = 0.04 at 300 K and 5 K and linear fit at high fields, (see text for details). (b) The variation of the ferromagnetic (FM) and paramagnetic (PM), components extracted from plots like (a) (as described in the text) shown as functions of the Zn composition (*x*). The inset shows the same two components with the magnetization in Bohr magnetons per Zn atom.

To separate the two components, we fitted the higher field data of the magnetization *versus* magnetic field (Fig. 7(a)) to the expression *M* = *M*_o_ + *eH* (where *e* is a constant) and from the linear fit the value of *M*_o_ was extracted, which will be referred to as the ferromagnetic component. The procedure is illustrated in Fig. 8(a). The values of *e*, the fitting constant representing the paramagnetic susceptibility, were obtained from the fits at *T* = 50 K and 300 K respectively. The ratio was found to be 5.2, which is close to the expected value of 6 for a purely paramagnetic behaviour, *χ* ∼ 1/*T*. The ferromagnetic component, *M*_o_, for each composition was obtained by the above procedure and then subtracted from the full moment measured at *H* = 10 kOe. The resultant value for each composition is referred to as paramagnetic component. The same procedure was also followed for the analysis of the data at lower temperatures, for Sn_1−*x*_Zn_*x*_O_2_ with *x* = 0.04 and *x* = 0.06.

The variation of both the magnetic components is shown as a function of the composition in the main part of Fig. 8(b). The inset shows the same two components with the magnetization in Bohr magnetons per zinc atom. Here the number of zinc atoms corresponds to the nominal concentration. We can see that the ferromagnetic moment per zinc atom has a maximum at *x* = 0.04 and is somewhat smaller for the *x* = 0.02, while it falls sharply at *x* = 0.06. We note that for *x* = 0.02 and 0.04 the lattice constant shows an expansion compared to *x* = 0.00, which indicates that zinc substitutes at Sn sites. The strong decline of the ferromagnetic moment/zinc atom for *x* = 0.06 coincides with the contraction of the lattice constant as shown in the XRD data.

Similarly the paramagnetic component/zinc atom (inset of Fig. 8(b)) is maximum at *x* = 0.04 and decreases very little for *x* = 0.06. However this contribution drops very strongly for Sn_1−*x*_Zn_*x*_O_2_ with both *x* = 0.02 and *x* = 0.10. This suggests that while the zinc dopants do not lead to a large ferromagnetic component for *x* > 0.04, they are still able to contribute strongly to the paramagnetic part for *x* = 0.06.

Consistent with the earlier discussion of the structural and electronic effects (see discussion of XPS data) it is possible that the observed variation of the ferromagnetic moment per zinc atom reflects a competing effect of two contributions, namely zinc dopants as hole contributors and stabilizers of Sn vacancies on the one hand, and oxygen vacancies as electron donors on the other.

For *x* = 0.02 and 0.04 the zinc atoms substitute for Sn but the number of oxygen vacancies is relatively small, leading to a larger ferromagnetic component. For higher Zn content it is possible that in addition to increasing the number of O vacancies, zinc occupies some sites other than those of Sn, *e.g.* O sites. Both features are expected to lead to a decline of the ferromagnetic behaviour. However the paramagnetic part can still be contributed to, as will be discussed next. There may be two different sources for this paramagnetic part. Firstly, the magnetic moment developed at sufficiently isolated defect sites (Zn_Sn_) will not lead to a stabilization of the FM^17^ but the paramagnetic contribution may still exist. Secondly we note that singly ionized oxygen vacancies can also yield a paramagnetic contribution. At room temperature all neutral V_O_ centres (an oxygen vacancy with two trapped electrons) are dissociated into a singly ionized oxygen vacancy 
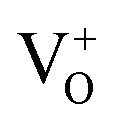
 (an oxygen vacancy with a single trapped electron) and a free electron. These singly charged oxygen vacancies have been reported to be paramagnetic.^47^ The decrease in this paramagnetic contribution at higher zinc concentration (*x* = 0.10) is then attributable to the recombination of O 2p holes with the trapped electron of the 
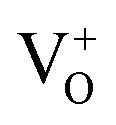
, converting it^47,48^ into a nonmagnetic 
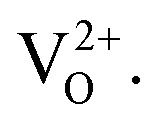
 We believe that the latter is the most plausible explanation for the variation of the paramagnetic moment with zinc concentration.

## Conclusions

Our measurements have shown, as have earlier studies,^6,21^ that incorporation of zinc in SnO_2_ nanoparticles enhances the ferromagnetic response very significantly but in a limited range of zinc concentrations. Our study represents an important step forward in understanding this phenomenon because it clarifies the particular defects that contribute to the magnetic moment. Structural and XPS studies confirm that the enhancement is strong in the region where zinc is incorporated substitutionally and the oxygen vacancy concentration is relatively small. This is understood in a picture where zinc substituting for Sn acts as a hole dopant for the O 2p bands, while oxygen vacancies counteract the effect by introducing electrons and reducing the hole concentration, thereby degrading the ferromagnetic response. There is a pronounced paramagnetic response, which we understand as originating from singly charged oxygen vacancies and possibly also from magnetic defects that are too far apart to stabilize ferromagnetism. Our studies further point out the role of morphology in stabilizing the moment-supporting defects. We find that the introduction of zinc leads to marked changes in the morphology of the nanoparticles. In particular, we identify that for the more strongly ferromagnetic compositions the nanoparticles have regular shaped structures with nanoneedles on their surfaces where the (110) and (101) planes are present. This is particularly significant in the sense that calculations^21^ have shown that ferromagnetic defects (V_Sn_) are energetically favoured on these surfaces. Hence there appears to be a correlation between the morphological structure and ferromagnetic behaviour *via* the anisotropic growth of nanostructures with surfaces that stabilize ferromagnetic defects. Thus ferromagnetism of the defects formed in Zn-doped SnO_2_ is thus a combination of three factors, namely stabilization of V_Sn_ and Zn_Sn_ defects; oxygen vacancies required for charge compensation and finally morphological variations that in turn affect both preceding factors by controlling the stabilization energies of various defects.

## Conflicts of interest

There are no conflicts of interest to declare.

## Supplementary Material
